# Business cycles’ correlation and systemic risk of the Japanese supplier-customer network

**DOI:** 10.1371/journal.pone.0186467

**Published:** 2017-10-23

**Authors:** Hazem Krichene, Abhijit Chakraborty, Hiroyasu Inoue, Yoshi Fujiwara

**Affiliations:** Graduate School of Simulation Studies, University of Hyogo, Japan; Universidad Rey Juan Carlos, SPAIN

## Abstract

This work aims to study and explain the business cycle correlations of the Japanese production network. We consider the supplier-customer network, which is a directed network representing the trading links between Japanese firms (links from suppliers to customers). The community structure of this network is determined by applying the Infomap algorithm. Each community is defined by its GDP and its associated business cycle. Business cycle correlations between communities are estimated based on copula theory. Then, based on firms’ attributes and network topology, these correlations are explained through linear econometric models. The results show strong evidence of business cycle correlations in the Japanese production network. A significant systemic risk is found for high negative or positive shocks. These correlations are explained mainly by the sector and by geographic similarities. Moreover, our results highlight the higher vulnerability of small communities and small firms, which is explained by the disassortative mixing of the production network.

## Introduction

With financial and economic globalization, trade relationships have become more complex. Consequently, different economies benefit from increases in their productivity and efficiency. However, the risk that “*modern*” economies collapse together has increased significantly. Over the past forty years, eight major crises have been triggered (e.g., the sovereign debt crisis in Latin American countries in 1982, the stock market crash in 1987, the Asian crisis in 1997–1998, and the global financial crisis in 2007–2008), representing an average of one crisis every five years. These successive crises changed the manner of thinking of many economists, who are looking for new tools and methods to understand the risks of “*modern*” economies.

Financial crises are always followed by economic slowdowns, which was reflected in GDP cycles (business cycles). [1] highlighted that the economics of Nordic countries recovered after 5–10 years, whereas the Japanese economic recession has continued for more than a quarter century. These systemic depressions are captured by persistent negative output gaps, which are reflected by negative business cycles (for additional explanations of the relationship between crisis and business cycles, see [2–4]). Accordingly, the propagation of positive and negative shocks in an economic system is captured by business cycle correlations between different groups (see [5]).

Many studies of business cycle correlations between countries have been conducted in order to explain the determinants of the contagion of economic recession and expansion. Imbs (2004) and Dai (2014) showed in [6, 7] that business cycle correlations between countries rise with trade volumes, capital flows and financial linkages. Moreover, [8] showed that industry-specific factors are significant for international business cycle correlations.

Most of these works were interested in the way the systemic risk spreads between countries. Few works presented intra-country analyses of how systemic risk spreads, i.e., of contagion between entities such as sectors or geographic regions. Understanding and explaining business cycle correlations within a country will identify the “*intrinsic*” systemic risk.

Artis and Okubo (2009) [9] studied Japanese business cycle correlations between prefectures based on a gravity model, and they observed significant correlations that are explained by the similarity of their GDP levels. This result was confirmed in [10] through an angular frequency analysis. By estimating a multiple equation econometric model, the same topic was studied in [11] to show that the business cycle correlations between U.S. states are explained by their trade flows and financial integration. (Similar results are shown in [12] using a wavelet analysis.) All these cited works are geography-based studies and assume that groups are defined by their regions (e.g., countries, prefectures, states). However, economic agents are heterogeneous and may form groups based on many factors other than their geographical positions. Thus, “*effective*” business cycle correlations could be better reflected by proper groups.

Gabaix (2011) [13] showed that general business cycle dynamics depend on the microscopic properties of the economic system. In fact, the author explained how modern economies are dominated by large firms, and idiosyncratic shocks to these firms can lead to nontrivial aggregate shocks that explain stylized facts about business cycles. Moreover, [14] argued that the presence of intersectoral input-output linkages and microeconomic idiosyncratic shocks may lead to aggregate GDP fluctuations. Thus, the aggregate business cycle of an economy can be approximated by the outcome of non-linear interactions between the most important firms. Following these results, in this paper, we will consider the production network of Japanese firms listed on the Tokyo Stock Exchange in order to uncover some stylized facts about the Japanese business cycle.

The Japanese production network is a directed network of firms, with links from suppliers to customers. The network is unweighted due to the lack of data. Suppliers are firms that produce capital goods, and customers are firms that produce final goods (firms can be both suppliers and customers). Therefore, the edges can be considered intersectoral input-output linkages as defined in [14] or channels of trade and capital flows as in the macroeconomic literature (see, e.g., [6]). As suggested in [13], the GDP of the economy will be approximated by the sum of the total sales of all firms. A very small sample of the global production network is selected, as will be explained in the data section. However, this sample is representative, with total sales of firms representing approximately 15% of the total sales of the production network. In addition, firms can be both suppliers and customers in the production network. Thus, the nature of risk depends on the contract terms. In fact, in the case of a contract with delayed payment, the supplier faces high risk. However, in the case of a contract with advance payment, the customer faces high risk. The absence of this information leads to risk in the production network that is borne by both sides (customers and suppliers). Accordingly, we consider a symmetric correlation matrix. In so doing, we ignore the edge direction of the network in the analysis of business cycle correlations.

As a novel contribution, we assume that business cycle correlations are related to the community structure of the production network. Communities are defined based on the Infomap algorithm (see [15]). Then, each community is specified by its own GDP, which is approximated by the sum of total sales of the community’s firms. By applying a Hodrick-Prescott filter, the business cycles of all communities are captured, and their correlations are modeled based on their bivariate joint distributions using copula theory. Copula theory is used to capture all the dependency structures of business cycles due to the non-normality of GDP fluctuations, as found in empirical works that provide stylized facts about GDP (see, e.g., [16]). Finally, the inter- and intra-community business cycle correlations are explained by different linear econometric models. These models will be fitted based on several economic variables and network topology variables.

This paper is organized as follows. Section 2 presents the data, some properties of the production network and its community structure. Section 3 provides an exposition of the methodology for estimating the business cycles and modeling the correlations based on copula theory. Then, section 4 presents different linear econometric models to understand and explain the observed business cycle correlations. Finally, section 5 provides some concluding remarks.

## The Japanese production network

The data for the Japanese production network are based on a 2016 study by Tokyo Shoko Research (TSR), which specifies for all pairs of firms whether there is relation from the supplier to the customer. The database contains more than one million firms with more than five million directed links.

To construct the GDP of the production network, some financial information is required, such as the total sales amount available on the PL statement of each firm. We used the Nikkei Digital Media database, which contains all financial information (e.g., balance sheets, PL) for firms listed on the Tokyo Stock Exchange from 1980 to 2012. After matching the two databases, only 3,199 firms were included in the production network, with a total of 20,415 directed links.

However, many firms were discarded due to missing financial data (those firms were created several years after 1980). Thus, the final number of firms included in the production network is 940 firms with 5,431 links. In the remainder of this section, different network properties are discussed, and then, the community structure based on the Infomap algorithm is explained.

### The production network topology

**Fig 1:**
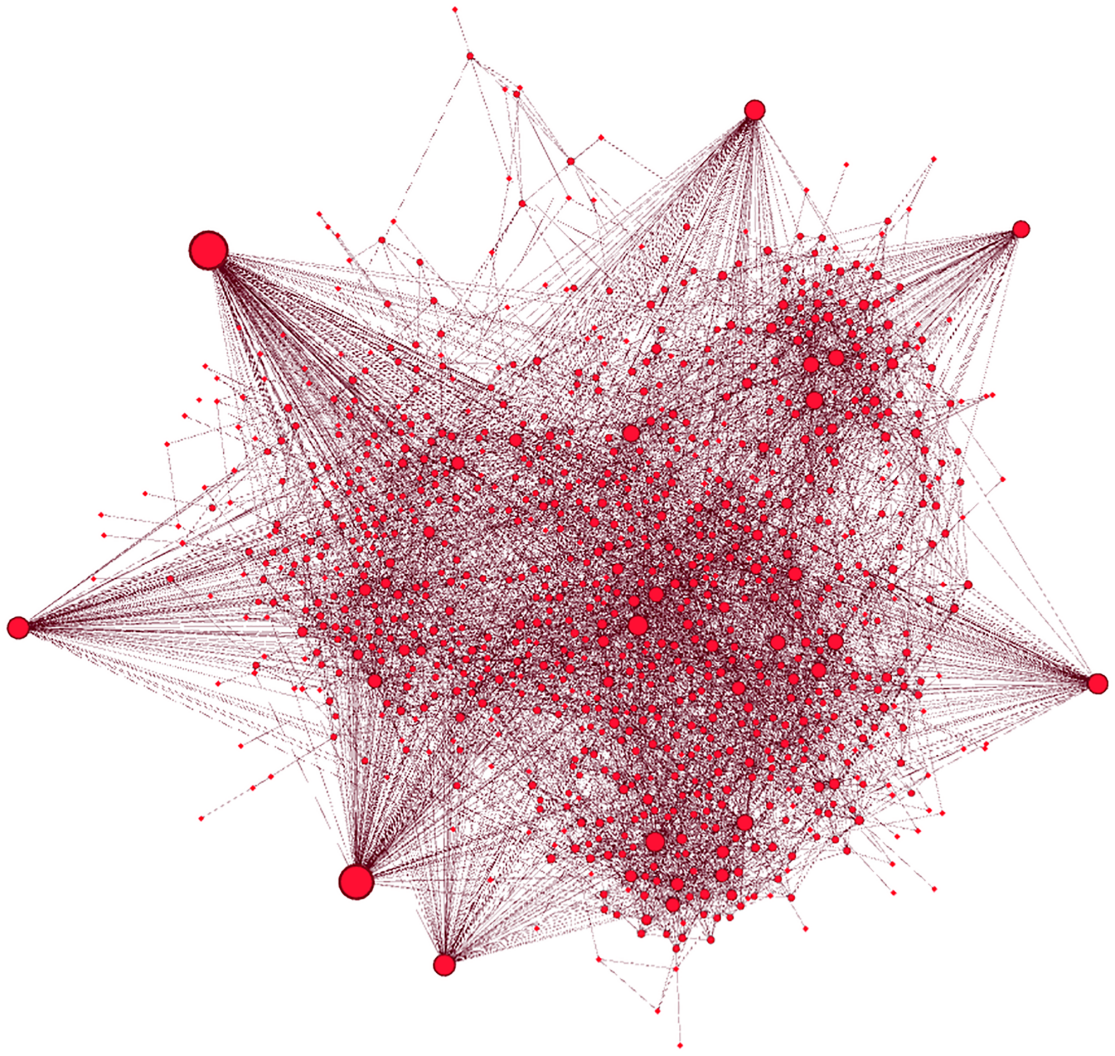
The Japanese supplier-customer network. It reveals the existence of a few large firms with high degrees and many small firms with low degrees.

**Fig 2:**
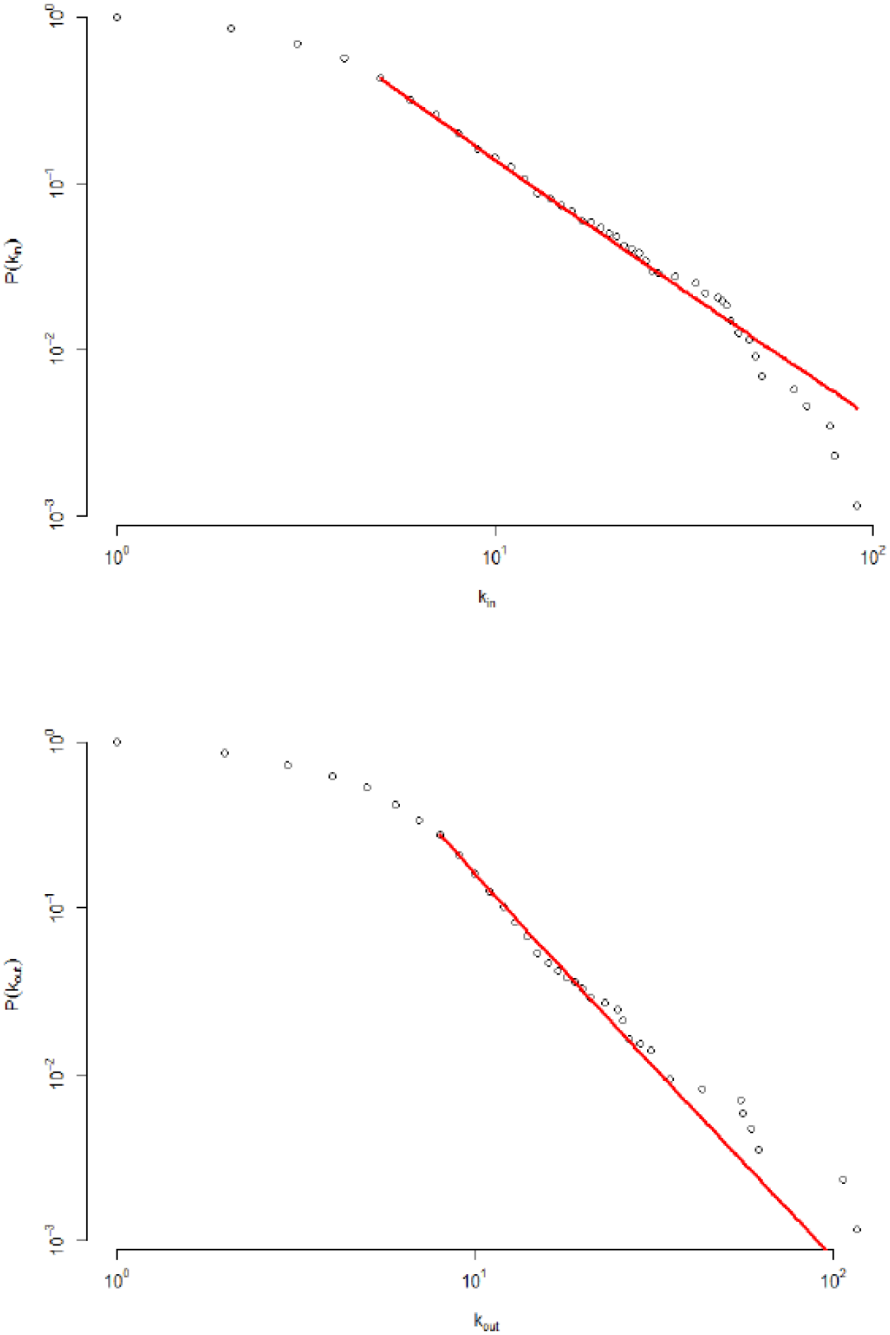
The degree distributions of the Japanese supplier-customer network. Both, the in and out degree distributions show scale-free behavior, with the respective power-law indexes $P\left(k_{in}\right)\propto k_{in}^{-2.52}$ and $P\left(k_{out}\right)\propto k_{out}^{-3.26}$.

Fig 1 illustrates the production network, which shows scale-free behavior. In fact, few firms have a high degree, which reflects their importance in the economy. This result is confirmed in Fig 2, which illustrates in and out degree distributions that exhibit power-law decay with indexes $P\left(k_{in}\right)\propto k_{in}^{-2.52}$ and $P\left(k_{out}\right)\propto k_{out}^{-3.26}$. This topology implies high heterogeneity, which confirms that idiosyncratic shocks to large firms have strong impacts on economic fluctuations, as explained in [13].

**Fig 3:**
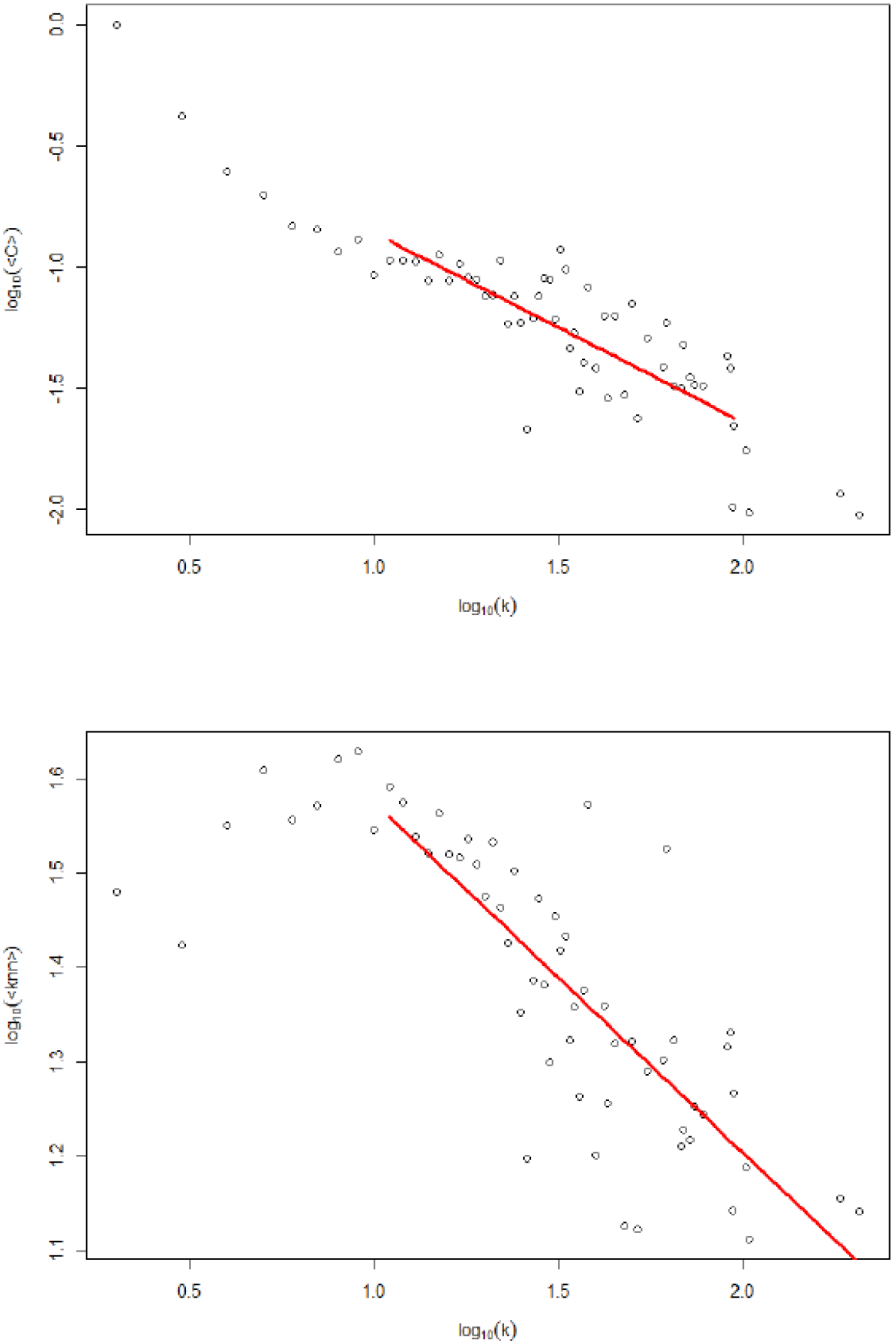
The relationship between average clustering, average nearest neighbors’ degrees and the total degree. Both figures show the disassortative topology of the Japanese production network *C*(*k*) ∝ *k*^−0.78^ and *P*(*k*_*nn*_) ∝ *k*^−0.39^.

Concerning the organization of the economy, the production network shows disassortative mixing (the assortativity coefficient, *r* = −0.18). Disassortative (assortative) mixing implies that firms with high degrees are more likely to be connected to firms with low (high) degrees. Indeed, in Fig 3, the clustering coefficient shows that the neighbors of the high-degree nodes are relatively less connected among themselves compared to the neighbors of low-degree nodes. Moreover, the average degree of the nearest neighbor shows that smaller nodes are more connected to larger nodes. Due to this topology, it is expected that small firms are more vulnerable during unstable economic periods to shocks to large firms.

### Communities of the production network

Community detection based on modularity maximization is based only on the network topology (see [17]). Thus, in order to extract the community structure based on the flow of capital goods between firms, we apply a community detection algorithm known as Infomap (for technical details, see [15]) to our directed network. In economic theory, firms produce using capital goods and labor, and economic growth is driven by innovation in capital goods. Thus, to extract communities from the production network, the links from suppliers to customers are considered.

Infomap is an information theoretic clustering method that can capture the complex structure of the network based on both topology and information diffusion dynamics. This algorithm uses a map equation in order to capture the hierarchical levels in the network and to determine how the communities are presented at each level.

Thus, to uncover the communities of the production network, capital flows between firms are approximated by using the Infomap procedure for random walkers, which is considered a diffusion process or information flow across the production network. The communities are the sub-units at which the random walker spends a longer time. In order to detect the community structure, the algorithm minimizes the code length average per step of the random walker.

**Fig 4:**
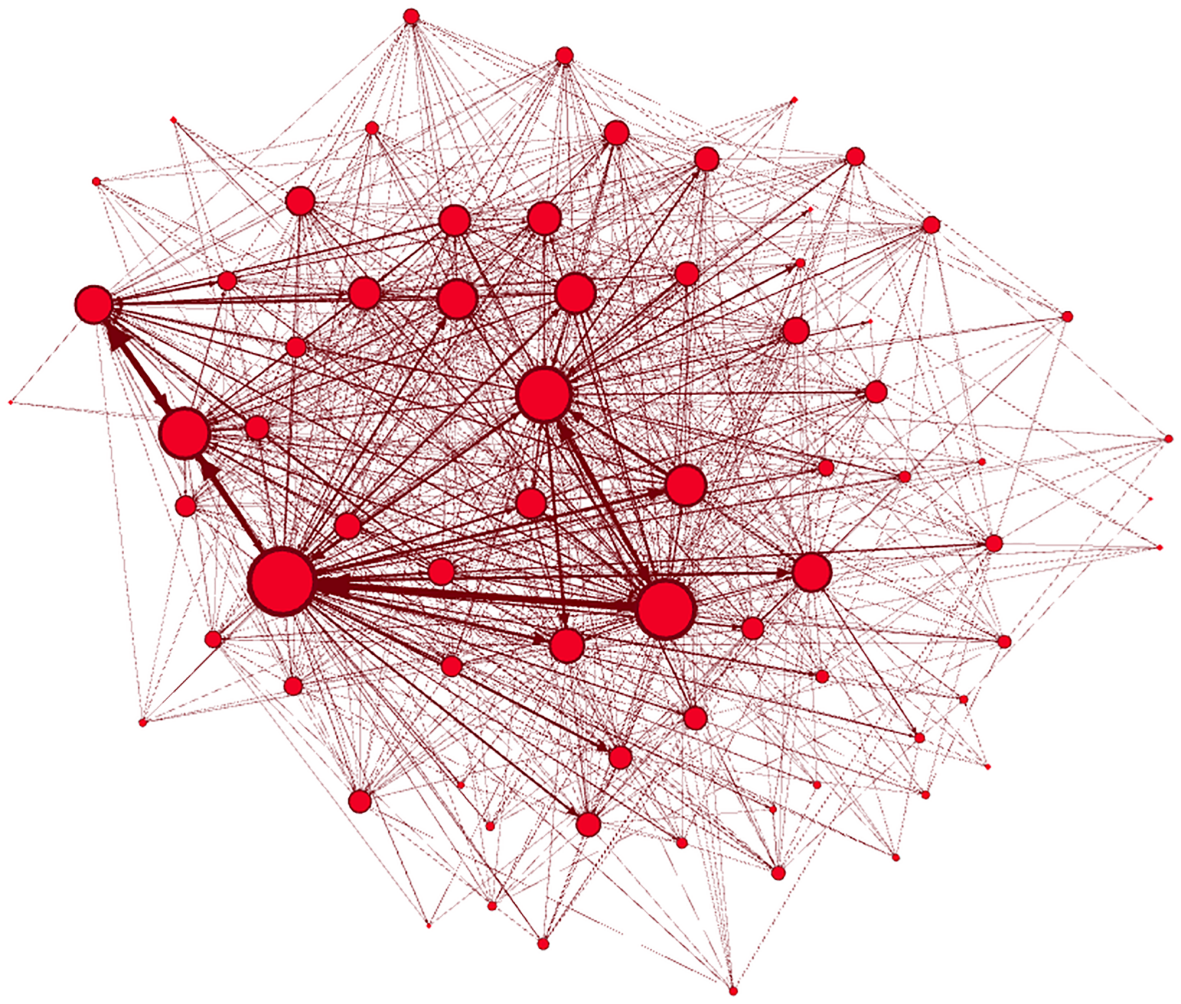
The coarse-grained network. It shows the scale-free topology of the network of communities.

**Fig 5:**
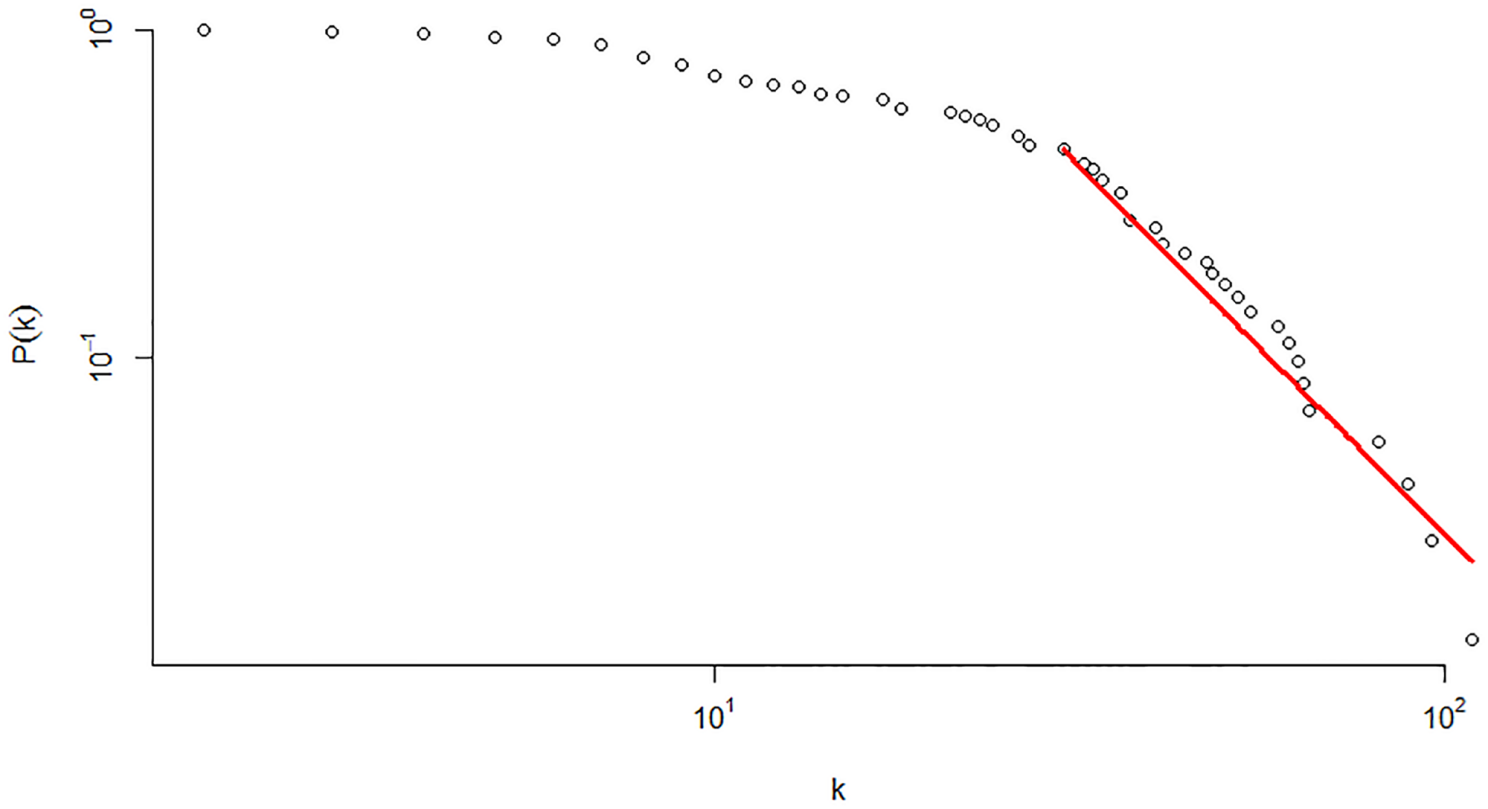
Total degree distribution of the coarse-grained network. It shows scale-free behavior with power-law decay of index *P*(*k*) ∝ *k*^−3.21^.

**Fig 6:**
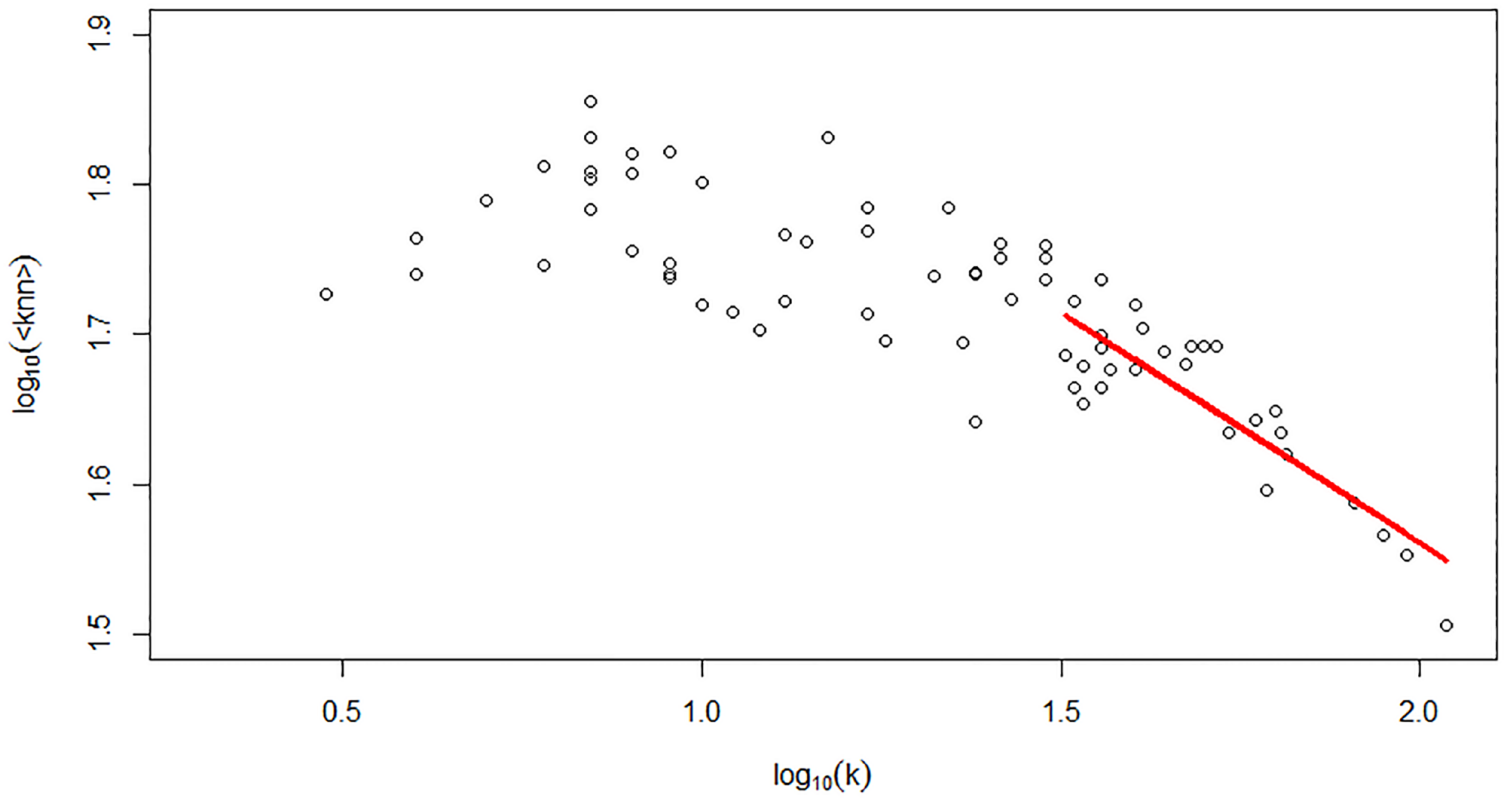
The relationship between the average nearest neighbors’ degrees and the total degree of the coarse-grained network. It shows the disassortative mixing of the coarse-grained network *P*(*k*_*nn*_) ∝ *k*^−0.31^.

The Infomap analysis of the directed production network reveals 73 modules with 1,028 inter-module links (Fig 4 depicts the topology of the coarse-grained network). To understand the community structure, the coarse-grained topology is studied, as shown in Figs 5 and 6, exposing the degree distribution and the average nearest neighbor degree. In Fig 5, the degree distribution shows power-law decay, with *P*(*k*) ∝ *k*^−3.21^ reflecting the existence of degree heterogeneity where a few “big” communities exist. In addition, in Fig 6, the decay of the average nearest neighbor degree with the total degree show disassortative mixing of the coarse-grained network, which is confirmed by the assortative coefficient *r* = −0.28. Finally, by the definition of the coarse-grained network, communities of smaller size (number of firms) have low connection, which implies that small communities have fewer trading partners. Thus, small communities have lower investment diversification, which could increase their risk of dependency.

## Assessment of Japanese business cycle correlations

This section provides an exposition of the econometric methodology used to capture the business cycles and to evaluate the correlations between communities and between firms.

### Econometric methodology and evaluation of business cycle correlations

Usually, GDP of a country is calculated based on its total income (consumption, sales, taxes, national income). The total sales of each firm is composed of its total income, paid taxes and employees’ salaries, which represent consumption. Therefore, we suppose that a community *α* with *N*_*α*_ firms defined by their total sales *S*_*i*_ for *i* ∈ [1, *N*_*α*_] has the following real GDP at time *t*:

$$\begin{array}{c} {\text{GDP}}_{\alpha ,t}=\frac{\sum_{i=1}^{N_{\alpha }}S_{i}}{D_{t}}\times 100, \end{array}$$ (1)

where *D*_*t*_ is the deflator of the economy for period *t*. Thus, for each community *α*, 33 annual observations are considered from 1980 to 2012. Our goal is to extract the fluctuations in GDP around its trend, which is known as the business cycle. Thus, the log of each *GDP*_*α*, *t*_ time series will be filtered according to the Hodrick-Prescott filter (see [18]). Let ${\hat{T}}_{\alpha ,t}$ be the estimated trend of *GDP*_*α*,*t*_. The estimated business cycle is given by ${\hat{X}}_{\alpha ,t}={\text{GDP}}_{\alpha ,t}-{\hat{T}}_{\alpha ,t}$. To simplify the notation, the estimated business cycle of community *α* will be denoted *X*_*α*_. The results presented hereafter were reproduced using two other filters (Baxter-King band-pass filter and Butterworth square-wave high-pass filter) to verify their robustness. We note that the constructed business cycle shows a high correlation with the actual Japanese business cycle observed between 1980 and 2012 and extracted using the Hodrick-Prescott filter (the regression gives a coefficient of 1).

Suppose that *X*_*α*_ and *X*_*β*_ are the business cycles of communities *α* and *β*, respectively. Their correlation is given by the bivariate joint distribution function F(*x*_*α*_, *x*_*β*_) = Pr(*X*_*α*_ ≤ *x*_*α*_, *X*_*β*_ ≤ *x*_*β*_). Let F_*α*_ and F_*β*_ be the respective continuous margins of *X*_*α*_ and *X*_*β*_. Then, *u*_*α*_ = F_*α*_(*x*_*α*_) and *u*_*β*_ = F_*β*_(*x*_*β*_) are uniform random variables according to the probability-integral transformation. Following the Sklar theorem, there exists a unique function C: [0, 1]^2^ ↦ [0, 1] such that:

$$\begin{array}{c} F\left(x_{\alpha },x_{\beta }\right)=C\left(u_{\alpha },u_{\beta };\Theta \right), \end{array}$$ (2)

where C is the bivariate copula function with parameter vector Θ. In this work, we consider the usual elliptical copulas (Gaussian and Student copulas) and several Archimedean copulas: Clayton, Gumbel, Frank, Joe, Clayton-Gumbel, Joe-Gumbel, Joe-Clayton and Joe-Frank. Some copulas are one-parameter copulas (Θ = *θ*_1_), such as the Clayton copula, while others are two-parameter copulas, such as the Joe-Gumbel copula (Θ = (*θ*_1_, *θ*_2_)); for more details, see [19].

To model the correlation structure through a copula, we start by testing the significance of the correlations between all bivariate business cycles (*X*_*α*_, *X*_*β*_). The independence test introduced by [20] based on the empirical Kendall $\hat{\tau }$ is applied. The test exploits asymptotic normality as follows:

$$\begin{array}{c} Q≔\sqrt{\frac{9N(N-1)}{2(2N+5)}}\cdot \left|\hat{\tau }\right|. \end{array}$$ (3)

The *p*—*value* of the null hypothesis of bivariate independence is given by:

$$\begin{array}{c} p-value=2\times (1-\Phi (Q)), \end{array}$$ (4)

where Φ is the standard univariate normal distribution function. Business cycles with non-rejected independence tests are considered non-synchronized. Then, the best fitting copula among the considered families is selected. In fact, for each pair of business cycles, the parameters of each copula are estimated based on the Inference Functions for Margins (IFM) method introduced by [21]. The copula with the lowest AIC value is selected. Based on this bivariate distribution function, the Kendall *τ* correlation between business cycles (*X*_*α*_, *X*_*β*_) is given by:

$$\begin{array}{c} \tau \left(X_{\alpha },X_{\beta }\right)=\int \int_{{\left[0,1\right]}^{2}}C\left(u_{\alpha },u_{\beta }\right)\;dC\left(u_{\alpha },u_{\beta }\right)\in \left[-1,1\right]. \end{array}$$ (5)

The Kendall *τ* measures the global correlation between 2 communities. Moreover, according to exogenous growth theory (see [22]), cycles are considered shocks producing short- and long-term fluctuations that propagate across the economy. The amplitudes of these exogenous shocks differ, and some extreme shocks can be observed due to the tent-shaped distribution of business cycles, as found in [23, 24]. Thus, to measure the correlations of these extreme movements between business cycles, upper and lower tail dependence ($\lambda_{\alpha ,\beta }^{U}$ and $\lambda_{\alpha ,\beta }^{L}$) are calculated as follows:

$$\begin{array}{c} \lambda_{\alpha ,\beta }^{U}=\lim_{v⟶1}\text{Pr}\left(X_{\alpha }>F_{\alpha }^{-1}\left(v\right)|X_{\beta }>F_{\beta }^{-1}\left(v\right)\right)=\lim_{v⟶1}\frac{1-2v+C(v,v)}{1-v}\in \left[0,1\right] \end{array}$$ (6)

$$\begin{array}{c} \lambda_{\alpha ,\beta }^{L}=\lim_{v⟶0}\text{Pr}\left(X_{\alpha }<F_{\alpha }^{-1}\left(v\right)|X_{\beta }<F_{\beta }^{-1}\left(v\right)\right)=\lim_{v⟶0}\frac{C(v,v)}{v}\in \left[0,1\right] \end{array}$$ (7)

Upper tail dependence for $\lambda_{\alpha ,\beta }^{U}\neq 0$ (lower tail dependence for $\lambda_{\alpha ,\beta }^{L}\neq 0$) shows the existence of economic boost (bust) risk between community *α* and community *β*. These non-normal shocks are expected during unstable periods (periods of crisis). Therefore, positive upper and lower tails reflect the probability of systemic risk in the economy, i.e., collective economic booms and busts.

### Business cycle correlations of the Japanese production network

**Fig 7:**
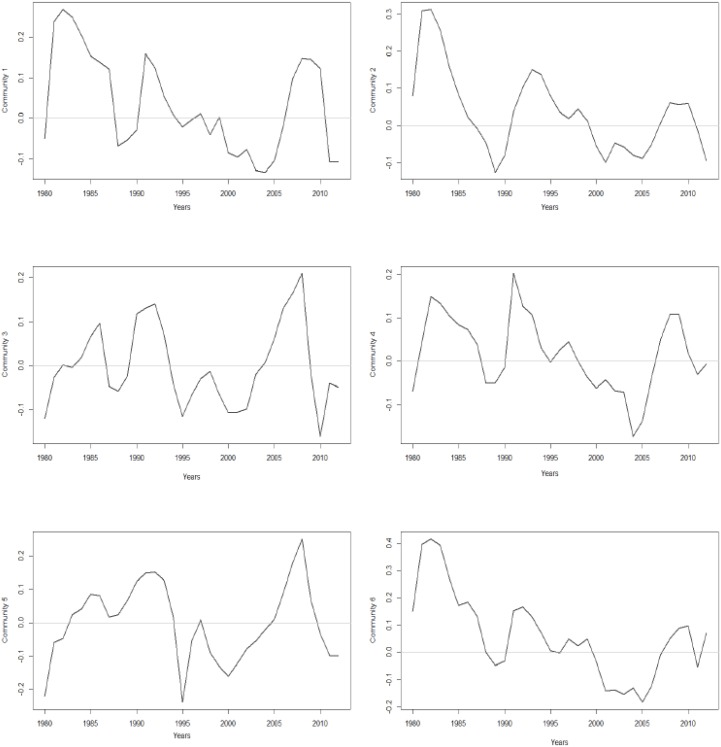
The business cycles of the six largest communities based on the Hodrick-Prescott filter and the de-trended GDP series. All communities show the presence of fluctuations based on boom and bust periods.

We recall that 73 communities were extracted from the Japanese production network, implying the existence of 2,628 bivariate business cycle correlations. Fig 7 shows the captured cycles of the six largest communities reflected in periods of economic recession and expansion.

**Fig 8:**
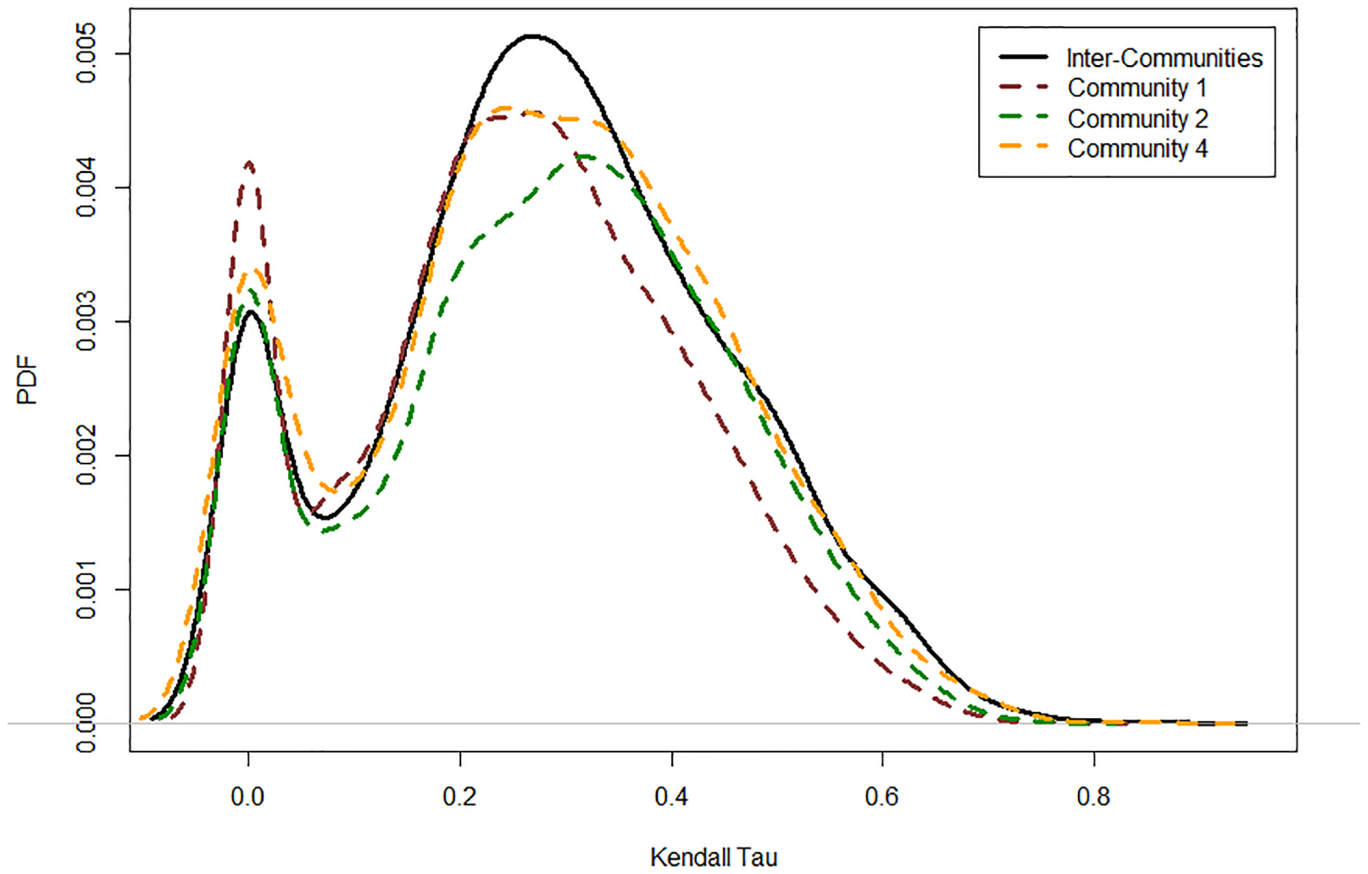
Probability density functions of the business cycle correlations in the Japanese production network. A comparison between the inter-community correlation and the within-community correlation (for the largest three communities).

**Fig 9:**
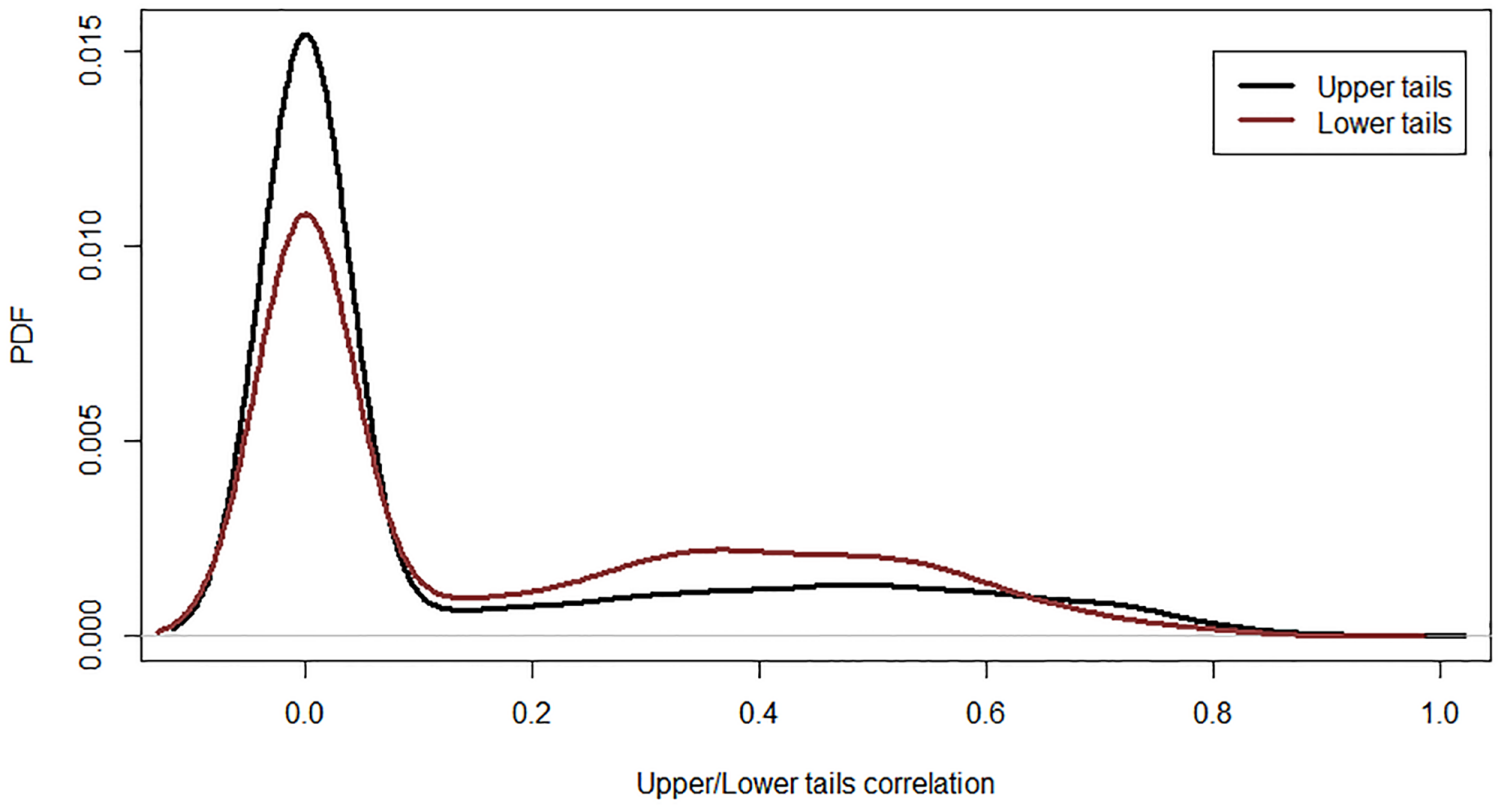
Probability density functions of boom and bust risk in the Japanese production network. They show the existence of significant systemic risk in the Japanese economy.

Based on the independence tests, 2,068 correlations are significant (560 bivariate joint distributions do not reject the null hypothesis of an independent copula). The inter-community correlations are calculated based on the Kendall *τ* and presented in Fig 8 (black curve). This result shows that the Japanese economy is interrelated through business cycle synchronization (as previously found by [9, 10]). Fig 8 compares the inter- and intra-community correlations. The intra-community correlations are calculated following the same copula methodology employed for the inter-community correlations and considers the filtered total sales of each firm. The results show that the inter- and intra-community correlations are very close. The similarity of the inter- and intra-community correlations is explained by the dense structure of the coarse-grained network (73 communities with 1,028 inter-modular links). Moreover, Fig 9 shows a significant presence of systemic risk in the Japanese production network through the positive values of upper and lower tail dependence. In fact, 33% (47%) of the estimated upper (lower) tails are significantly different from zero. These correlations indicate that positive or negative shocks may propagate across the economy.

We note that these correlations are not driven by large firms. In fact, we discarded the largest firms from our sample (firms with weights in terms of total sales of more than 1%). These 20 firms represent 2% of the total sample size. Following the same procedure, the business cycle correlations of the two samples (with and without large firms) are very similar (without large firms, the mean of correlation between communities is 0.28, with a standard deviation of 0.15).

## Explanation of the inter- and intra-community correlations: Multiple linear econometric models

The determinants of inter- and intra-community business cycle correlations are studied in this section. Understanding the origins of these dependencies is crucial to protecting the economy from disastrous crises due to the risk that shocks spread.

### Inter-community correlations

We will start this part by motivating and explaining the proposed econometric models. The results of the linear least squares estimation are exposed and analyzed.

#### Some determinants of business cycle correlations between communities

Our aim is to determine which economic variables explain the business cycle correlations between communities. The economic system is highly complex, and many phenomena and variables (e.g., demography, politics, social atmosphere) interfere simultaneously and reproduce the observed macro patterns. However, many simplifications and assumptions are needed to consider the most important variables in order to explain these correlations.

Some works related business cycle correlations to financial features (e.g., trading volume, capital flows), such as [6, 7, 25]. These features were considered by construction in our model. In fact, business cycle correlations are defined based on the production network linkages that reflect the capital flows and trading links between firms. However, information about volumes is missed, and the network is kept unweighted instead of introducing additional assumptions about the trading volume, which would change the results for the community structure and the business cycle correlations.

Other works showed the importance of the sector factor as a determinant of business cycle correlations. [8, 10, 14] showed the importance of, respectively, intersectoral input-output linkages, business cycle synchronization between sectors in Japan and industry-specific factors in international business cycle co-movements. To model the sector factor, we considered the 82 major group sectors in Japan. Then, for each community, the sector’s distribution was calculated. The sector similarity between two communities *α* and *β* was calculated based on the Jensen-Shannon distance (see [26]) of each sector distribution as follows:

$$\begin{array}{c} {\text{JS}}_{\alpha ,\beta }^{sector}=\frac{1}{2}[\sum_{s=1}^{sectors}P_{\alpha }\left(s\right)\cdot \text{log}(\frac{P_{\alpha }\left(s\right)}{P_{\alpha }\left(s\right)+P_{\beta }\left(s\right)})+\sum_{s=1}^{sectors}P_{\beta }\left(s\right)\cdot \text{log}(\frac{P_{\beta }\left(s\right)}{P_{\beta }\left(s\right)+P_{\alpha }\left(s\right)})]. \end{array}$$ (8)

*P*_*α*_(*s*) and *P*_*β*_(*s*) represent the probability of sector *s* in communities *α* and *β*, respectively. The smaller the distance, the higher the sector similarity between communities.

In [25, 27, 28], it was shown that the business cycle correlation between two groups (countries or regions) is decreasing with the geographical distance. To model the geographic factor, the 47 Japanese prefectures were considered. For each community, the prefecture distribution was determined. Then, the Jensen-Shannon distance was applied to measure the geographic similarity between two communities as follows:

$$\begin{array}{c} {\text{JS}}_{\alpha ,\beta }^{geo}=\frac{1}{2}[\sum_{s=1}^{prefectures}P_{\alpha }\left(s\right)\cdot \text{log}(\frac{P_{\alpha }\left(s\right)}{P_{\alpha }\left(s\right)+P_{\beta }\left(s\right)})+\sum_{s=1}^{prefectures}P_{\beta }\left(s\right)\cdot \text{log}(\frac{P_{\beta }\left(s\right)}{P_{\beta }\left(s\right)+P_{\alpha }\left(s\right)})]. \end{array}$$ (9)

The GDP similarity between groups was considered an important factor to explain the business cycle correlations. Indeed, in [9, 25, 28], it was found that groups with similar GDP vales are more likely to be correlated. To define GDP similarity, we start by rescaling the GDP of each community onto the interval [0, 1], ${\text{GDP}}_{\alpha }^{r}=\frac{\text{log}{\left(\text{GDP}\right)}_{\alpha }}{\max_{\beta \in \text{Communities}}\left(\text{log}{\left(\text{GDP}\right)}_{\beta }\right)}$. Then, the GDP distance $D_{\alpha ,\beta }^{\text{GDP}}$ is calculated by:

$$\begin{array}{c} D_{\alpha ,\beta }^{\text{GDP}}=\left|{\text{GDP}}_{\alpha }^{r}-{\text{GDP}}_{\beta }^{r}\right|. \end{array}$$ (10)

Moreover, in the financial crisis literature, the weight of institutions (e.g., banks, insurance companies) is important in defining the systemic risk. This idea is reflected in the “*Too Big Too Fail*” theory: a shock to a larger economic agent is more likely to spread across the economy (see, e.g., [29, 30]). Similarly, in this work, we proposed to measure the impact of community size on the business cycle correlations. The community size *S*_*α*_ is the total number of firms in the community, which is rescaled onto the interval [0, 1], $S_{\alpha }^{r}=\frac{S_{\alpha }}{\max_{\beta \in \text{Communities}}\left(S_{\beta }\right)}$. Then, community size similarity is calculated by the following distance:

$$\begin{array}{c} D_{\alpha ,\beta }^{S}=\left|S_{\alpha }^{r}-S_{\beta }^{r}\right|. \end{array}$$ (11)

Based on these variables, we aim to explain the Japanese business cycle correlation structure and some determinants of the observed systemic risk. The explanation will be achieved by estimating the following linear econometric models of the Kendall *τ* correlation and upper and lower tail dependence:

$$\begin{array}{c} \tau_{\alpha ,\beta }=\beta_{1}{\text{JS}}_{\alpha ,\beta }^{sector}+\beta_{2}{\text{JS}}_{\alpha ,\beta }^{geo}+\beta_{3}D_{\alpha ,\beta }^{\text{GDP}}+\beta_{4}D_{\alpha ,\beta }^{S}+\text{intercept}+\epsilon_{\alpha ,\beta } \end{array}$$ (12)

$$\begin{array}{c} \lambda_{\alpha ,\beta }^{U}=\beta_{1}{\text{JS}}_{\alpha ,\beta }^{sector}+\beta_{2}{\text{JS}}_{\alpha ,\beta }^{geo}+\beta_{3}D_{\alpha ,\beta }^{\text{GDP}}+\beta_{4}D_{\alpha ,\beta }^{S}+\text{intercept}+\epsilon_{\alpha ,\beta } \end{array}$$ (13)

$$\begin{array}{c} \lambda_{\alpha ,\beta }^{L}=\beta_{1}{\text{JS}}_{\alpha ,\beta }^{sector}+\beta_{2}{\text{JS}}_{\alpha ,\beta }^{geo}+\beta_{3}D_{\alpha ,\beta }^{\text{GDP}}+\beta_{4}D_{\alpha ,\beta }^{S}+\text{intercept}+\epsilon_{\alpha ,\beta }. \end{array}$$ (14)

#### Comments on and analysis of the estimation results

**Fig 10:**
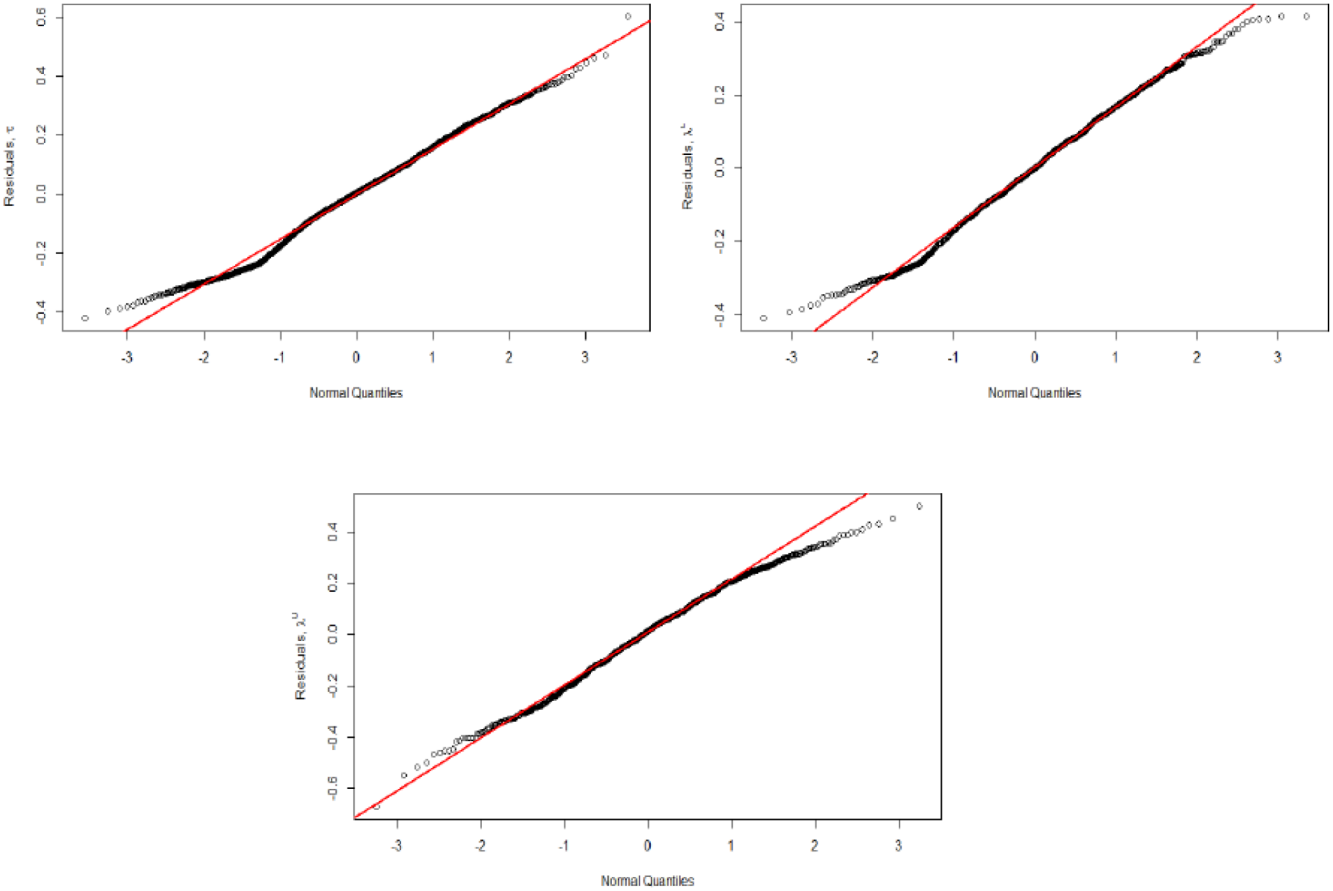
QQ-plots of residuals of the three estimated linear econometric models. Validation of the homoscedasticity assumption.

The estimation results are presented in Table 1. The QQ-plots in Fig 10 confirm the homoscedasticity condition for the three estimated linear models. Concerning the Kendall *τ* estimation, the results show that the community size impact is non-significant. However, the significant negative values of sector and geographic distance indicate that the higher the sector and geographic similarities, the higher the business cycle correlations. This finding confirms previous results in the literature such as [10, 28]. Surprisingly, the positive significant coefficient of the GDP distance indicates that the lower the GDP similarities, the higher the business cycle correlations, which contradicts previous works such as [9, 25, 28]. This result is explained by the network disassortative mixing, which implies that small communities are more likely to be connected to large communities. These small communities have low degrees that reflect their low investment diversification strategy, which increases their dependence on large communities.

**Table 1:** Results of the three estimated linear econometric models.

	*τ*	λ^*U*^	λ^*L*^
${\text{JS}}_{\alpha ,\beta }^{sector}$	−0.29*** (0.03)	−0.36*** (0.08)	−0.29*** (0.05)
${\text{JS}}_{\alpha ,\beta }^{geo}$	−0.17*** (0.02)	−0.04 (0.05)	−0.09* (0.03)
$D_{\alpha ,\beta }^{\text{GDP}}$	0.13** (0.04)	0.05 (0.08)	0.17*** (0.06)
$D_{\alpha ,\beta }^{S}$	−0.004 (0.04)	0.10 (0.08)	−0.04 (0.05)
intercept	0.58*** (0.03)	0.72*** (0.06)	0.37*** (0.04)

This table aims to explain the determinants of Japanese business cycle correlations and the origins of collective economic booms and busts.

*, ** and *** indicate significant values at the 10%, 5% and 1% levels, respectively.

By analyzing the positive extreme co-movements λ^*U*^ in Table 1, we find that only the sector homophily factor is significant. This result means that communities with similar sector distributions are more likely to exhibit extreme positive business cycle correlation. However, the negative extreme co-movements λ^*L*^ show that economic recession depends on both sector and location homophilies. Thus, communities with similar sector and location distributions have a higher risk of facing a common economic depression. Finally, community GDP heterophily shows a significant positive relationship, which implies that the greater the dissimilarity in the communities’ GDP, the higher the extreme negative business cycle correlations. This result is explained by the disassortative mixing of the network and confirms previous explanations of the higher vulnerability of small communities.

**Fig 11:**
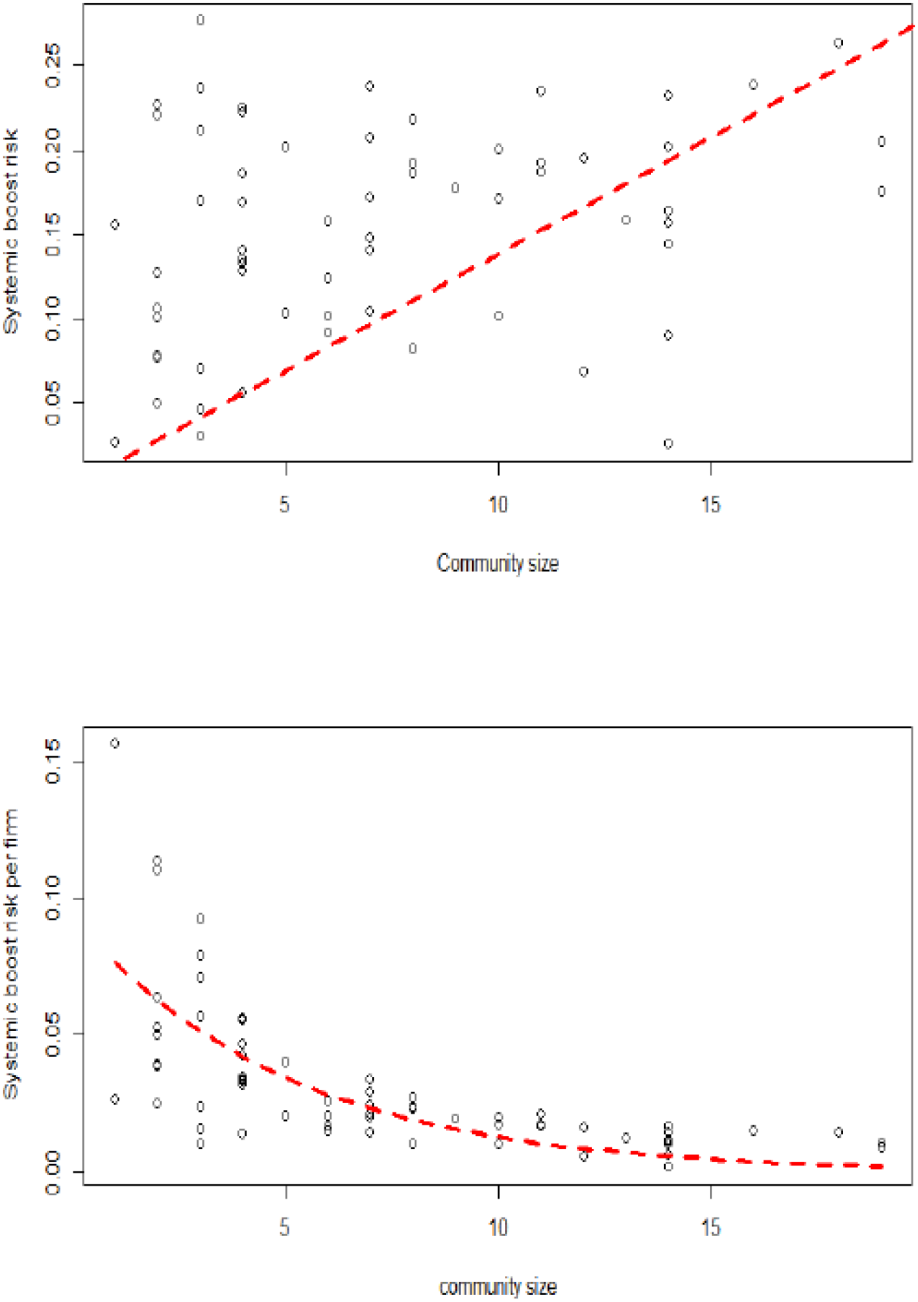
The relationship between upper tail dependence (systemic boom risk) and community size (the largest communities with more than 20 firms are not considered). (Left) the global systemic boom risk; (right) the systemic boom risk per firm.

**Fig 12:**
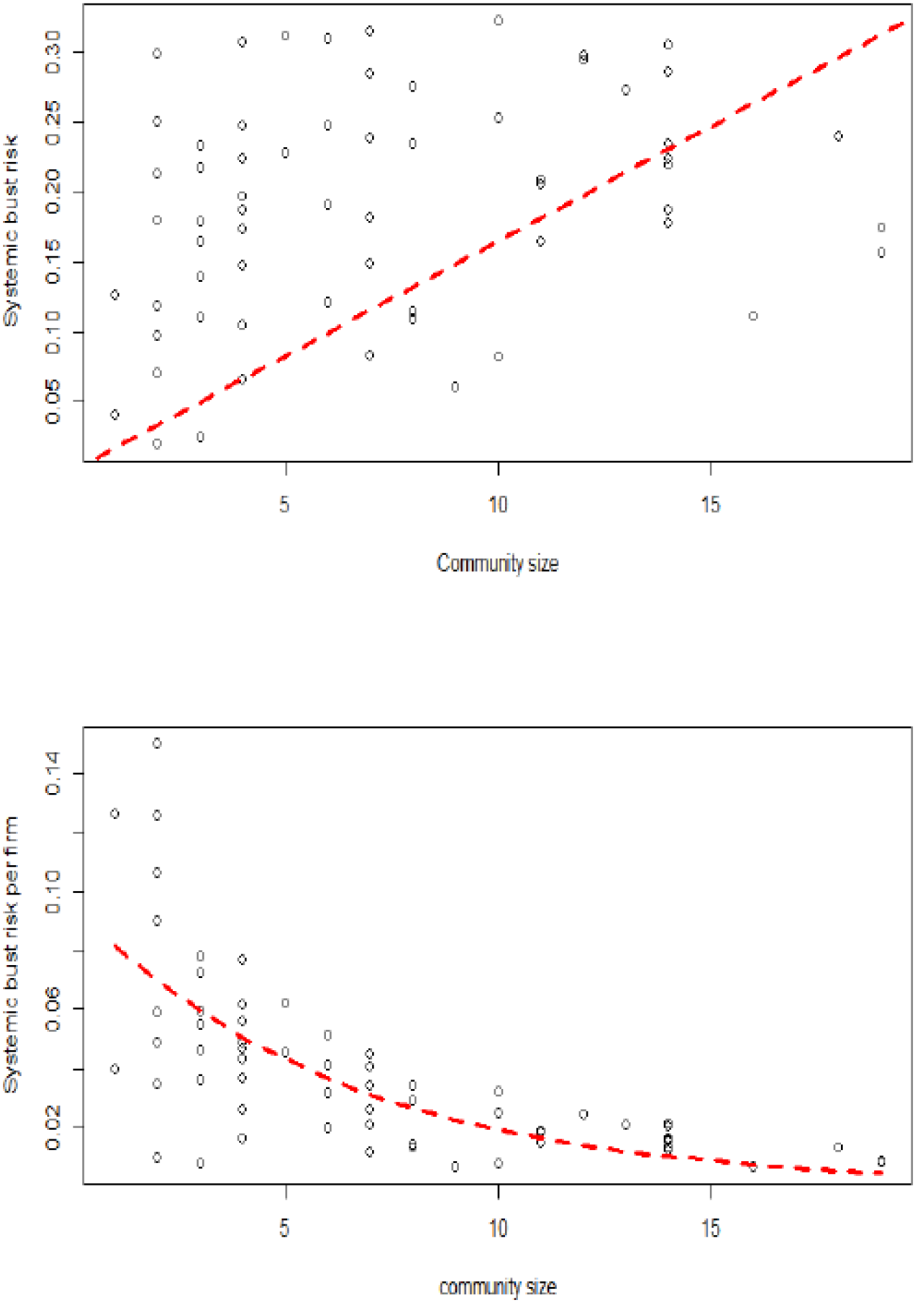
The relationship between lower tail dependence (systemic bust risk) and community size (the largest communities with more than 20 firms are not considered). (Left) the global systemic bust risk; (right) the systemic bust risk per firm.

This finding is explained through Figs 11 and 12. These figures represent the community size and the systemic boom and bust risks for each community *α* ($\sum_{\beta }\lambda_{\alpha ,\beta }^{U}$ and $\sum_{\beta }\lambda_{\alpha ,\beta }^{L}$). On the left side of Figs 11 and 12, it is shown that most communities have higher risk compared to their size. Moreover, it is clear that the smaller the community size, the higher the systemic risk, which confirms the vulnerability of small communities. On the right side of Figs 11 and 12, it is shown that the systemic risk borne, on average, by firms in each community is decreasing with the community size, which confirms the higher vulnerability of small communities. The decrease in systemic boom (bust) risk per firm with respect to community size was approximated by an exponential function obtained based on a non-linear least squares estimation (see [31]): 0.09 × exp(−0.20 × communitysize) (0.09 × exp(−0.16 × communitysize)).

### Intra-community correlations

Fig 8 showed evidence of business cycle correlations at the intra-community level. In this part, we aim to explain the determinants of these dependencies in the production network topology.

#### Some determinants of business cycle correlations within communities

The business cycle correlations at the intra-community level are studied hereafter based on the properties of the firms in the production network. Three axes are analyzed in this part: the firms’ degree homophily, the firms’ clustering homophily, and the distance between firms.

The firms’ social homophily is studied through the degree and clustering. To understand how the degrees of two firms *i* and *j* affect their correlation, the degree homophily ${\text{Degree}}_{ij}^{homophily}$ measure is used. After rescaling the firms’ degrees onto the interval [0, 1] (each firm degree is divided by the total network degrees), the measure is defined as follows:

$$\begin{array}{c} {\text{Degree}}_{ij}^{homophily}=\left|{\text{Degree}}_{i}^{r}-{\text{Degree}}_{j}^{r}\right|. \end{array}$$ (15)

Following the same methodology, the clustering homophily is given as follows:

$$\begin{array}{c} {\text{Clustering}}_{ij}^{homophily}=\left|{\text{Clustering}}_{i}^{r}-{\text{Clustering}}_{j}^{r}\right|. \end{array}$$ (16)

In [32], the authors have shown that correlations increase in the production network between firms with lower shortest paths. Thus, to model this effect, in our analysis, we consider the shortest path between firm *i* and firm *j*, denoted by Path_*ij*_. Therefore, the following main equation will be estimated:

$$\begin{array}{c} \tau_{ij}=\beta_{1}{\text{Degree}}_{ij}^{homophily}+\beta_{2}{\text{Path}}_{ij}+\beta_{3}{\text{Clustering}}_{ij}^{homophily}+\text{intercept}+\epsilon_{ij}. \end{array}$$ (17)

#### Comments on and analysis of the estimation results

**Fig 13:**
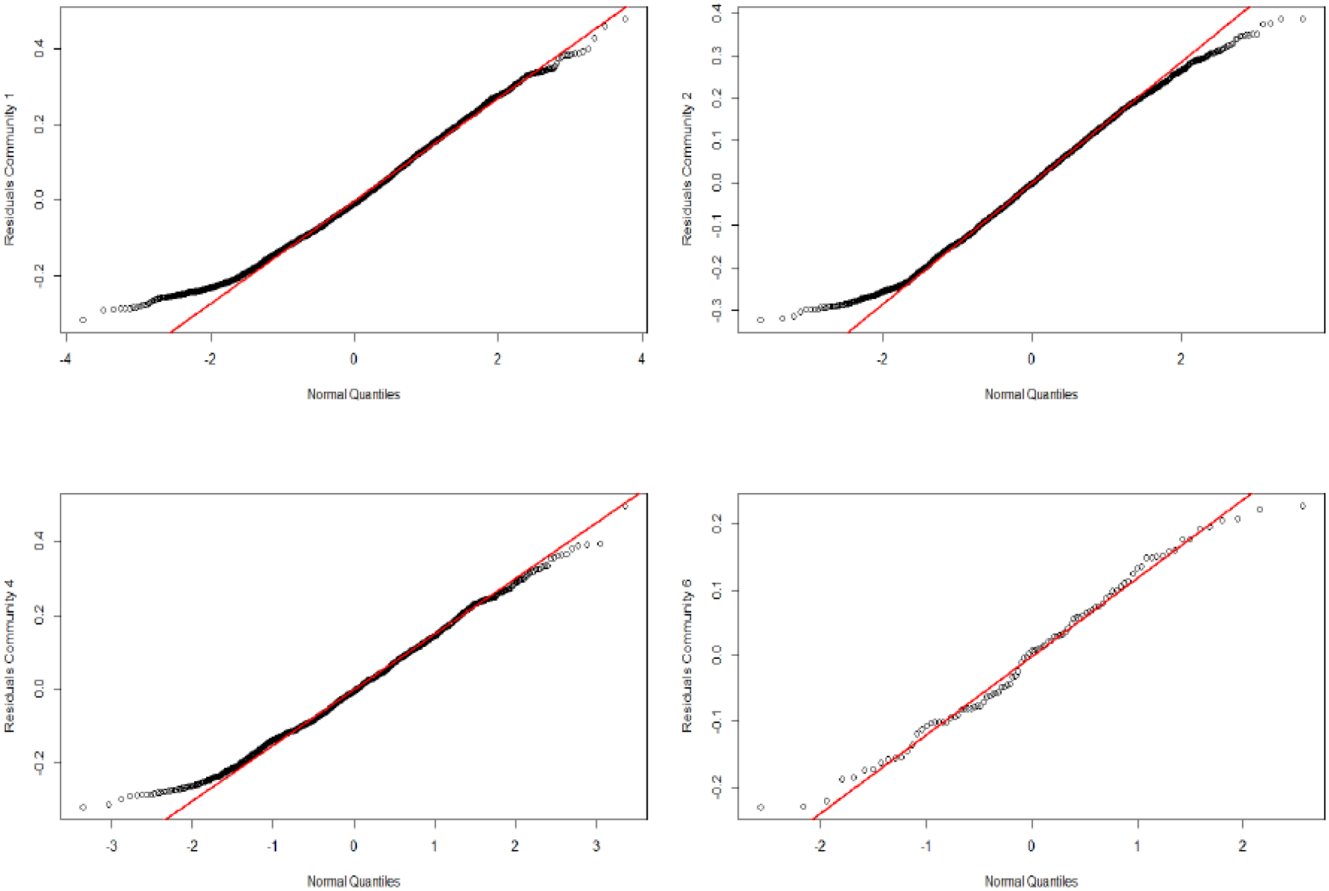
QQ-plots of residuals of the four estimated linear econometric models. Validation of the homoscedasticity assumption.

To understand the intra-community business cycle correlations, linear least squares estimation of Eq 17 was applied to four communities: the three largest communities (community 1: 123 firms, community 2: 94 firms and community 4: 62 firms) and a medium-sized community (community 6: 23 firms). Other communities are not considered due to their small size (10, on average), which would yield only 11 observations with which to estimate each coefficient. The results are reported in Table 2. The QQ-plots in Fig 13 confirm the homoscedasticity condition for the four estimated linear models.

**Table 2:** Results of the linear econometric model in Eq 17.

	*τ*_1_	*τ*_2_	*τ*_4_	*τ*_6_
${\text{Degree}}_{ij}^{homophily}$	0.07*** (0.01)	0.02* (0.01)	0.01 (0.02)	0.09 (0.08)
Path_*ij*_	−0.06*** (0.01)	−0.22*** (0.02)	−0.03 (0.03)	−0.15 (0.11)
${\text{Clustering}}_{ij}^{homophily}$	−0.02** (0.01)	−0.04** (0.01)	−0.04** (0.02)	0.03 (0.03)
intercept	0.29*** (0.01)	0.38*** (0.01)	0.31*** (0.02)	0.28*** (0.01)

This table aims to explain the determinants of Japanese business cycle correlations within communities.

*, ** and *** indicate significant values at the 10%, 5% and 1% levels, respectively.

All variables are significant for communities 1 and 2. First, the results show that the correlation increases with degree heterophily. As found previously in the inter-community analysis, disassortative mixing implies that large firms are connected to small firms that have few trade partners, which increases their dependence. Moreover, the shortest path shows that firms with closer network distances have more correlations, as found in [32]. In addition, it is shown that firms with similar clustering levels are more likely to be dependent.

However, these results are significant only for the largest communities. Based on Table 2, communities 4 and 6 show non-significant results. This means that the production network effect cannot explain the correlations between firms in small communities. However, due to the limited number of observed communities, we cannot draw conclusions about the causal effect of community size or explain their internal correlations based on the network topology.

## Discussion and conclusion

Understanding how different economic agents, such as banks and firms, are correlated is of high importance to measuring the systemic risk of a country. This topic interested many authors who studied business cycle correlations (see, e.g., [6, 25]). Regarding the Japanese economy, some works studied this topic through pre-defined groups based on sector or geographic classifications ([9, 10]). However, as explained in other works ([13, 14]), we highlighted the importance of considering the real microscopic structure of the economy in order to analyze the collective dynamics of business cycles.

In a new contribution, we considered the Japanese supplier-customer network to classify groups based on the community structure determined by the Infomap algorithm. These communities reflect the proper group-based structure of the Japanese production network, which is based on both the sector and geography. Then, the business cycle of each community was estimated based on the total sales of firms to study the inter- and intra-community correlations modeled using copula theory.

First, the results showed evidence of significant business cycle correlations at the inter- and intra-community levels. These correlations were very similar, indicating that dependence risks are significant at the firm and community levels. The dependence between communities is explained by the dense structure of the coarse-grained network, which showed power-law decay and disassortative mixing.

We found that the inter-community business cycle correlations increase with sector and geographic similarities. However, we showed contradictory results from those of some previous works that argued that correlations between groups increase with GDP similarity. In fact, we showed that due to the disassortative mixing of the network, small communities are linked to large communities, which increases their dependence. This vulnerability of small communities was confirmed by analyzing the systemic risk based on the upper and lower tails with copula modeling. This finding highlights the importance of considering the proper group structure instead of pre-defined groups.

The intra-community business cycle correlations were studied through four selected communities. For the largest communities in the network, we showed significant positive impacts of the shortest distance and clustering homophily on business cycle correlations. Moreover, the impact of disassortative mixing was confirmed at the firm level, where correlations increase with the dissimilarity of firms’ degrees. However, we found non-significant business cycle correlations for the small communities based on their internal structure.

This work identified some stylized facts about the business cycles in the Japanese production network. However, further research can be conducted in order to deepen and enrich the results: Which microscopic components can explain the business cycle correlations? How can an economic agent’s vulnerability be shown based on its internal structure? The first topic can be studied by constructing an artificial economy based on a real network and reproducing these stylized facts about business cycles through some microscopic patterns. The second topic can be considered by simulating how shocks spread across the production network based on firm or community properties.
